# Cortical facilitation of tactile afferents during the preparation of a body weight transfer when standing on a biomimetic surface

**DOI:** 10.3389/fneur.2023.1175667

**Published:** 2023-06-19

**Authors:** Chloé Sutter, Alix Moinon, Livia Felicetti, Francesco Massi, Jean Blouin, Laurence Mouchnino

**Affiliations:** ^1^Laboratoire de Neurosciences Cognitives, FR 3C, CNRS, Aix Marseille Université, Marseille, France; ^2^Department of Mechanical and Aerospace Engineering, Sapienza University of Rome, Rome, Italy; ^3^LAMCOS, INSA Lyon, CNRS, UMR5259, Université Lyon, Villeurbanne, France; ^4^Institut Universitaire de France, Paris, France

**Keywords:** plantar sole afferents, voluntary movement, sensory gating, biomimetic surface, balance, EEG, somatosensory evoked potentials (SEPs), gamma oscillations

## Abstract

Self-generated movement shapes tactile perception, but few studies have investigated the brain mechanisms involved in the processing of the mechanical signals related to the static and transient skin deformations generated by forces and pressures exerted between the foot skin and the standing surface. We recently found that standing on a biomimetic surface (i.e., inspired by the characteristics of mechanoreceptors and skin dermatoglyphics), that magnified skin–surface interaction, increased the sensory flow to the somatosensory cortex and improved balance control compared to standing on control (e.g., smooth) surfaces. In this study, we tested whether the well-known sensory suppression that occurs during movements is alleviated when the tactile afferent signal becomes relevant with the use of a biomimetic surface. Eyes-closed participants *(n* = 25) self-stimulated their foot cutaneous receptors by shifting their body weight toward one of their legs while standing on either a biomimetic or a control (smooth) surface. In a control task, similar forces were exerted on the surfaces (i.e., similar skin–surface interaction) by passive translations of the surfaces. Sensory gating was assessed by measuring the amplitude of the somatosensory-evoked potential over the vertex (SEP, recorded by EEG). Significantly larger and shorter SEPs were found when participants stood on the biomimetic surface. This was observed whether the forces exerted on the surface were self-generated or passively generated. Contrary to our prediction, we found that the sensory attenuation related to the self-generated movement did not significantly differ between the biomimetic and control surfaces. However, we observed an increase in gamma activity (30–50 Hz) over centroparietal regions during the preparation phase of the weight shift only when participants stood on the biomimetic surface. This result might suggest that gamma-band oscillations play an important functional role in processing behaviorally relevant stimuli during the early stages of body weight transfer.

## Introduction

Inputs from the foot cutaneous receptors provide information on the body's position in space while standing or walking. Our brain has the ability to control the amount of these cutaneous cues that will be processed by filtering out or increasing their transmission. Since the seminal study of Chapin and Woodward (1) in rats, and later ones in humans (2–5), the transmission of cutaneous inputs from the periphery to the cortical level has been probed by assessing the brain areas' sensitivity to electric skin stimulation. Sensory inputs can undergo the so-called movement-related sensory gating (6, 7). This suppression or attenuation of sensory inputs corresponds to a top-down filtering of the afferent information and is hypothesized to be linked to motor prediction: an efferent signal from motor areas canceling out the predicted sensory reafferences that arise during voluntary movements (8, 9). It has been proposed that this sensory gating's role is to differentiate between sensations created by one's own movements and sensations resulting from external stimuli thereby assigning greater weight to less predictable external sensory inputs (9, 10). While the movement is accompanied by sensory gating, it is also generally acknowledged that when sensory information is relevant to behavior, the movement-related sensory gating can be partially alleviated (11–13). For example, using electric stimulation of the tibial nerve containing mainly afferents from the sole of the foot, Duysens et al. (3) demonstrated that during locomotion, there was a phase-dependent modulation of the sensory suppression. The gating was partly alleviated before footfall, that is likely to anticipate the need for cutaneous information for the forthcoming foot placement. The use of electrical stimulation ensures that stimulation remains rather constant throughout the movement, but this has a drawback since the stimulation is without informational contents and non-selective for the types of cutaneous afferents activated. Hence, the richness of information provided by the four functionally distinct types of tactile sensors encoding skin mechanical deformation is lost (i.e., information on the time course, magnitude, direction, and spatial distribution of contact forces, skin stretches, and the friction between contacted surfaces and the skin; see [(14, 15) for reviews].

On the basis of previous behavioral and electrophysiological studies which show that movement-related gating can be partly alleviated when sensory information is relevant to the task (3, 13), we tested whether the enhancement of relevant information for balance control enabled by specific skin-biomimetic surface contact interaction would help counteract the movement-related sensory suppression. To test this hypothesis, we recorded the cortical responses to skin/surface interaction (i.e., somatosensory-evoked potential, SEP) because it represents the amount of sensory transmission and the early sensory processes (16–18). This was done when standing participants voluntarily shifted their body weight laterally toward one foot (hereafter named “active task”). The body weight shift is known to be initiated by exerting forces onto the supporting surface [e.g., (19)], which in turn stimulates the cutaneous receptors of the plantar sole. We compared the SEP recorded when the participants stood either on a biomimetic or a smooth surface (i.e., surfaces enhancing or impoverishing relevant tactile information, respectively). Indeed, a recent study showed faster and greater responses of the somatosensory cortex when the participants were resting upright on a translating biomimetic surface than on a smooth surface (20). We also measured the SEP in a passive task, where the participants were standing motionless and similar forces as in the active task were passively generated by translating the surfaces under the participants' feet (i.e., similar skin–surface contact interaction as in the active task). Since there was no voluntary movement of the participant that could have induced sensory suppression (at the time of the SEP measurement), this passive task allowed us to normalize, for both types of surfaces, the amount of sensory gating during the self-generated movement. In most of the studies, the SEP was found to be reduced by ~55 to 70% with respect to the SEP measured in a “resting condition” [e.g., (3, 5, 7, 21)]. We expected that executing the voluntary body weight transfer on a biomimetic surface providing relevant tactile cues might suppress or at least lessen the sensory gating observed during movement execution.

Moreover, to move the whole body safely while standing, the brain must be informed about the body's position relative to the support surface prior to body motion (22–25). The foot sole's cutaneous receptors are thought to contribute to this information because, while the body is motionless, the foot sole undergoes pressure variations due to postural sways that stimulate the cutaneous receptors (26). For instance, Mouchnino et al. (5) [see also (27)] observed that the transmission of foot cutaneous inputs to the cortex (following electric stimulation of the foot sole) was facilitated during the preparation stage of a step movement (~700 ms before motor execution) compared to a standing condition without step preparation. This sensory facilitation during the preparation of the upcoming stepping movement could contribute to building up an accurate representation of the body's position in space. We hypothesized that the biomimetic surface that enhances the stimulation of foot mechanoreceptors should increase the efficiency of the sensory processing during the preparation of the body weight shift. To test this hypothesis, we analyzed gamma event-related synchronization (ERS) during the preparation phase preceding the body weight transfer toward the supporting leg. Indeed, previous studies show that movement preparation is accompanied by an increased synchronization of cyclical fluctuations in neuronal excitability across populations of neurons over the sensorimotor cortex in the gamma bandwidth (30–50 Hz), before the start of the movement (28, 29). The current knowledge on the functional significance of gamma event-related synchronization (ERS) is largely related to the preparation of the movement characteristics [e.g., increased ERS was scaled with movement distance and peak velocity (29)]. However, converging lines of observations point to the critical role of gamma ERS to increase the efficiency of sensory processing during the movement preparation period. Indeed, Tatti et al. (29) found greater synchronization over the posterior regions known to integrate motor signals with proprioceptive and visual information (30–32), and Palmer et al.'s study (33) showed a relationship between increased gamma ERS and enhanced perception for the forthcoming force reproduction (i.e., fewer errors). Based on the abovementioned studies, we expected that participants who are preparing to shift their body weight to one leg will show greater gamma activity in the somatosensory and parietal cortices when they are standing on a biomimetic surface compared to a smooth surface.

## Materials and methods

### Participants and task

Twenty-five participants (13 women) without any known neurological and motor disorders participated in the experiment (mean age 23 ± 2 years, mean weight 68 ± 12 kg). All participants gave their written informed consent to take part in this study, which conformed to the ethical standards set out in the Declaration of Helsinki and which was approved by the research ethics committee CERSTAPS (IRB00012476-2021-09-12-140).

**Figure 1:**
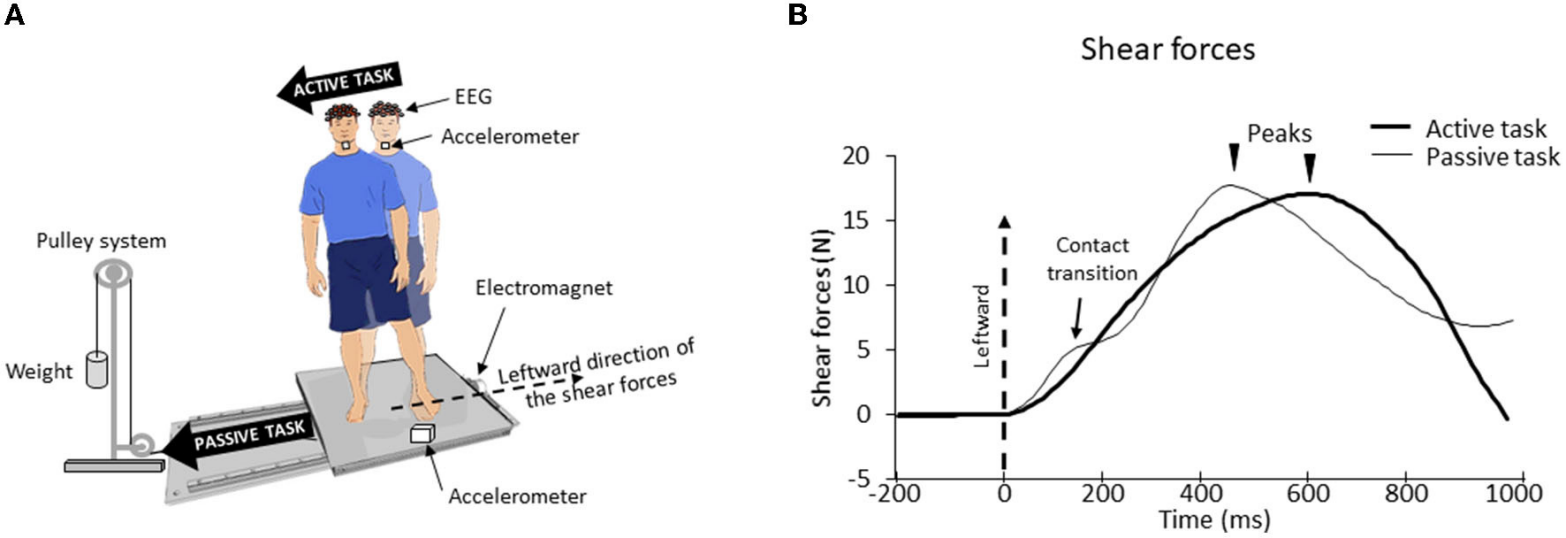
**(A)** Experimental setup. The participant stood barefoot on a force platform which, on deactivation of the electromagnet, would undergo a translation due to gravity loading. **(B)** Mean lateral forces for one representative participant for both surfaces (biomimetic, smooth) during active and passive tasks. The time 0 (vertical dotted line) corresponds to the onset of the shear forces change. For the passive task, the first peak force (here smoothed due to the average) corresponds to the maximal extensibility of the skin under the feet until the frictional force (i.e., shear force) can no longer resist the sliding leading to transient variations of the local strain distribution (“skin–surface contact transitions”). Afterward, a second force peak occurred, corresponding to a voluntary postural reaction.

Participants were requested to stand barefoot with their feet at a natural distance apart on different types of surfaces (see below), fixed in the middle of a movable force platform. They wore a safety harness attached to the ceiling. The feet's position was kept constant across the experimental session. We used a setup employed in previous studies for stimulating foot tactile afferents (34). The platform was positioned on two guide rails (Bosh Rexroth) with a ball-bearing system to reduce friction. The platform was held stationary by an electromagnet and could be translated to the right by deactivating the electromagnet. A cable attached to this platform (at the opposite side of the electromagnet) was connected at the other end to a pulley system with a load fixed to its extremity (Figure 1A). The load was adapted to the participants' weight, such that switching off the electromagnet allowed the platform to accelerate to the participants' right, without endangering their balance.

At the start of a trial, the participants looked at a fixation point which was positioned at the eye level, 2 m directly in front of them. They were asked to close their eyes upon receiving verbal instructions on the upcoming task and to remain still. The instruction indicated one of these three tasks: active (40 trials), passive (40 trials), and stationary (10 trials). The experimental session, therefore, included 90 trials which were randomly distributed.

During the active task, participants had to shift their body weight toward their right foot, while keeping both feet on the surface. This body weight shift was initiated by propulsive forces (vigorous pressure onto the ground) in the leftward direction (Figure 1B). The participants were instructed to perform their body movement ~2 s after receiving the instruction on the upcoming task. For the passive task, the electromagnet was deactivated ~2 s after this instruction. This released the platform which translated to the right of the participants, thereby triggering passive shear forces in the leftward direction. The participants were asked to remain upright during the translations. The stationary trials were used to analyze vibration and EEG signals (see below). During these trials, the participants had to remain still during 20 s.

**Figure 2:**
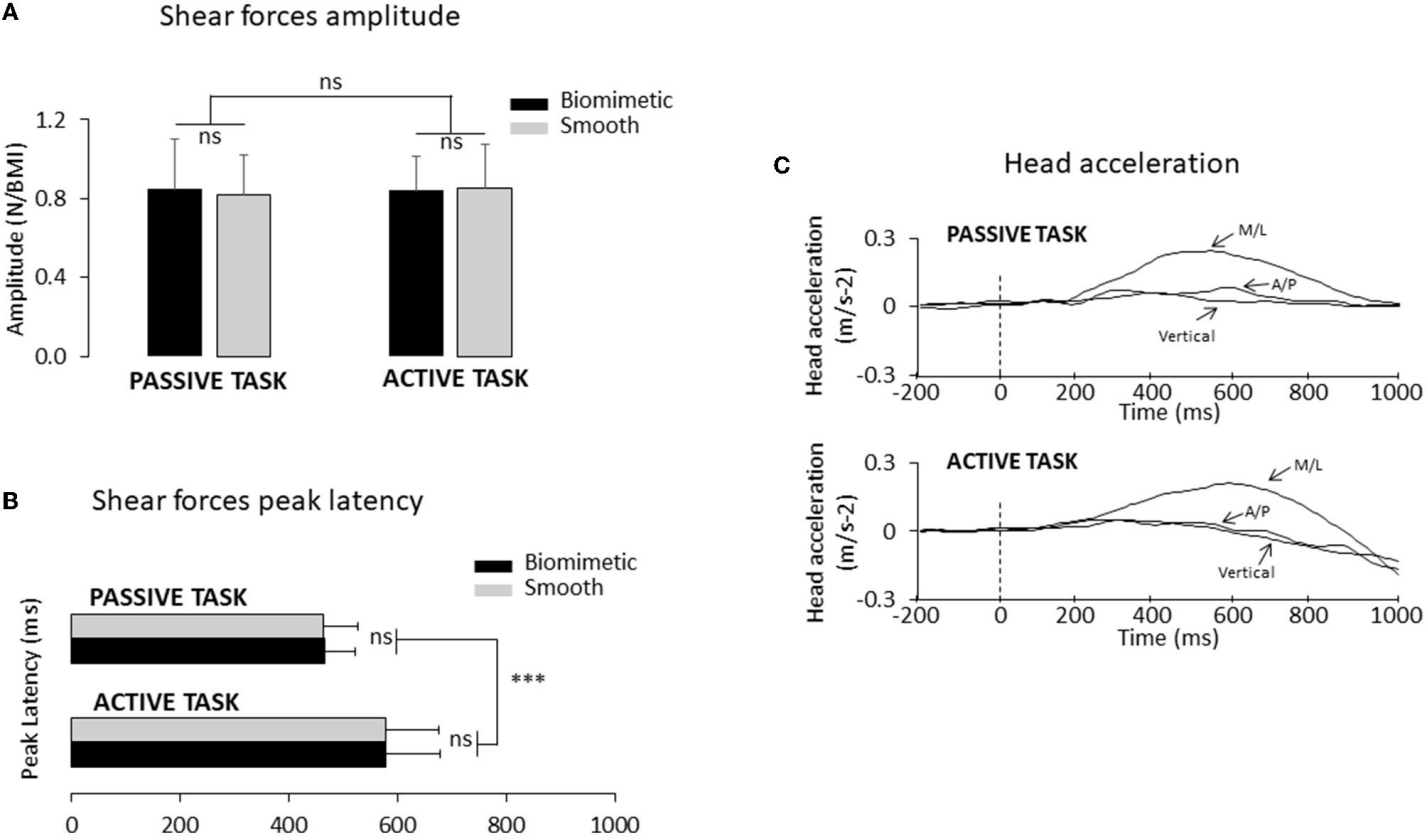
**(A)** Mean mediolateral forces amplitude normalized to the BMI for all participants (*n* = 25). Error bars represent standard deviation across participants; ns, not significant. **(B)** Mean time to peak duration for all participants (***p < 0.001). **(C)** Mean head acceleration computed for one representative participant using the biomimetic and the smooth surfaces, in the passive task (top panel) and in the active task (bottom panel). *Three* axes of the head acceleration: M/L corresponds to the mediolateral axis, A/P corresponds to the anteroposterior axis, and vertical corresponds to the vertical axis. The time 0 (vertical dotted line) corresponds to the onset of the shear forces change.

To make the shear forces comparable between the active and passive tasks (Figure 1B), before the experimental session, we measured for each participant the lateral forces that were passively elicited by the platform translation in three trials. Afterward, we asked the participants to shift their body weight in a rapid and accurate manner to reproduce the same forces as those produced in these “passive” trials. The experimenter controlled for the potential difference (e.g., forces rise time and amplitude) between these forces. Verbal corrective instructions were provided to the participant if necessary. The participants needed ~3 to 10 trials to match the initial passive forces (i.e., those evoking the SEPs) in a satisfactory manner. During these trials, the participants were standing on the surface (i.e., biomimetic or smooth, see below for their description) on which they were standing at the start of their experimental session (see below). Note that this training session, which lasted ~3 min, unlikely altered the sensory gating [see (35), for evidence of unaltered SEP gating during repetitive practice]. The analyses showed that, for both types of surfaces, the amplitude of the shear forces exerted by participants on the platform in the active task was fairly similar to those recorded in the passive task (see Figure 2A). An ANOVA indicated that the peak shear forces were not significantly affected by the tasks (*F*_1.24_ = 0.74; *p* = 0.40) although the peak force was reached later for the active task (578 ± 98 ms) than for the passive task (465 ± 60 ms) (significant task effect, *F*_1, 24_ = 28.77; *p* = 1.7^*^10^−5^, Figure 2B). Finally, neither the peak amplitudes (*F*_1, 24_ = 0.21; *p* = 0.65) nor their latencies (*F*_1, 24_ = 1.19^*^10^−5^; *p* = 0.99) were significantly affected by the type of surface on which the participants were standing.

### Surfaces

Participants stood on two different surfaces which were glued onto the platform: a biomimetic surface and a smooth surface. These surfaces were created with a 3D printer (Ultimaker 2+) using biopolymer thermoplastic (polylactic acid, PLA). Three characteristics were selected to build the biomimetic surface: shape, spatial period, and depth of the ridges. Circular shapes were inspired by both the shape of the tactile receptors' fields that demonstrate a preferential skin strain axis and the orientation of this axis, which was not the same for all units (36, 37) and the circular forms of the dermatoglyphics (38). For instance, Scheibert et al. (39) showed that when scanning a surface with a fake finger, friction-induced vibrations (FIVs), whose characteristics depend not only on the surface texture but also on the fingerprints (40–42), were amplified when the fake finger had the main geometrical characteristics of human fingerprints compared to when it had a smooth surface (i.e., no ridges). The power spectrum issued from the finger exploration showed an amplification of the signal around the frequency pertaining to the optimal sensitivity range of the Pacinian receptors [i.e., ranging between 100 and 300 Hz (43)].

The spatial period of the biomimetic surface corresponded to the distance between the center of adjacent receptive fields of the mechanoreceptors (43). Finally, the depth of the valley between the ridges was computed from what we know based on finger surface exploration (44, 45) and balance maintenance literature (22). A smooth surface, also printed in PLA but without any designed patterns, was used as a control surface.

### Behavioral recordings and analyses

The ground reaction forces and moments were recorded with an AMTI force platform (60 × 120 cm, Advanced Mechanical Technology Inc., USA) at a sampling rate of 1,024 Hz and low pass filter (Butterworth 4th-order, 10 Hz cutoff frequency). In our study, the shear forces were analyzed only along the mediolateral (ML) axis as they were in the same direction as the platform translation and body weight transfer. The shear forces were normalized to each participant's body mass index (BMI) which takes into account both their height and their weight.

Head acceleration was recorded at a frequency of 1,024 Hz using a triaxial accelerometer (4630 Model: Measurement Specialties, USA) placed on the participants' chin. We measured the delay between the shear forces change onset and lateral head acceleration to verify if vestibular stimulation occurred well after the mechanoreceptor's stimulation. The vestibular stimulation threshold was defined when the lateral head acceleration exceeded 0.048 m.s^−2^ [i.e., threshold for vestibular stimulation (46)]. We paid particular attention to the two other axes (i.e., vertical and anteroposterior axis) before the platform displacement to be sure that no head tilt occurred along these axes (Figure 2C).

Another accelerometer (PCB 352A24, PCB Piezotronics, Inc.) was glued with wax onto the participant's right first toenail to measure the friction-induced vibrations (FIV), i.e., the vibrations induced by the transient local phenomena (local sticking, sliding, and detachments) occurring at the skin–surface contact interface. Eight out of twenty-five participants were excluded from the accelerometric analyses due to noisy accelerometer signals. Providing information related to the frequencies and intensities of the foot vibration, this highly sensitive monoaxial accelerometer (sensitivity of 100 mV/g and measurement range of ±50 g peak) allows us to get an estimate of the stimulation of the tactile receptors of the foot sole. From the measurement of these FIVs, we computed the power spectral density (PSD). These power spectra describe the distribution of power into frequency components composing the signal and give information on the frequencies excited during the frictional contact interaction when the foot skin interacts with the surface. The sampling frequency of the acquisition system was 1,024 Hz with an average analysis time window of 183 ± 72 ms. One hundred twenty-eight samples were used for the calculation of the NFFT (i.e., a type of Fourier transform). The period considered was defined from the shear forces onset to the breakdown in the curve observed during the passive task (i.e., contact transition) as this period was considered to be at the origin of the response of the somatosensory cortex to the stimulation of the cutaneous receptors of the foot sole [see Figure 1B and (20)]. The same period was used for the active task even though no such breakdown was observed.

Data transmitted by the accelerometer fixed on the toenail could also include noise (e.g., electromagnet activation/deactivation) or contain signals that arose from vibrations that were not linked to the skin–surface interaction (friction of the platform on the ball-bearing system). To test for such possibilities, we ran a series of trials in which the participants stood on either the biomimetic or the smooth surface while the accelerometer was glued on either of the surfaces or directly on the platform. The signals were recorded when the platform was held stationary with the electromagnet and when it translated sideways. To translate the platform, we either used the activation/deactivation of the magnet (as in the main experiment) or manually held the platform (rather than using the electromagnet) before releasing it. This series of tests allowed us to isolate the vibrations that are generated by the experimental setup from those generated by skin–surface interaction. These tests showed that the ball-bearing system produced very negligible vibrations and that the vibrations recorded at the level of the biomimetic and smooth surfaces were very similar. However, a clear peak emerged at ~100 Hz when using the deactivation of the electromagnet to trigger the platform translation (**Figure 5C**). This peak was absent during the stationary trials and when the translations were manually triggered. We conducted a t-test on the power of the peak (which is evoked only by the deactivation of the electromagnet) against a reference value of 100 Hz. This test revealed that the emerging peak of nearly 100 Hz is too closely related to the deactivation of the electromagnet because no significant difference appeared from the reference value (*t*_13_ = 1.29; *p* = 0.22). As a result, this peak has not been treated as a characteristic vibrational signature of the smooth surface. For each surface, we calculated a mean PSD of the 15 test trials. The time window for this analysis was similar to the one mentioned above, and it is the one that corresponds to the initial skin–surface interaction (20). Because the low frequencies, under 20 Hz, are mainly due to the macroscopic vibrations (overall motion) of the mechanical system composed by the platform and the participant's body, and because these low frequencies are far from the sensitive range of cutaneous receptors, we considered for the analyses the (20–500 Hz) bandwidth frequency vibrations propagating through tissues [see (14) for a review]. For example, Pacini endings (fast-adapting type II) are extremely sensitive to mechanical transient high-frequency vibrations (~40–400 Hz), and Meissner endings (fast-adapting type I) are sensitive to dynamic skin deformation with a peak of response at ~30–40 Hz.

For the FIV analyses, we normalized (with subtraction) the PSD measured in both the active and passive tasks with respect to the PSD measured in the stationary condition (i.e., no movement during 20 s). Furthermore, for the passive task, to eliminate the noise associated with the deactivation of the electromagnet, we subtracted, for each surface, the mean PSD which was calculated in the 15 test trials (see above).

### Electrophysiological recordings and analyses

Electroencephalographic (EEG) activity was continuously recorded from 64 Ag/AgCl surface electrodes embedded in an elastic cap (BioSemi ActiveTwo system: BioSemi). Specific to the BioSemi system, “ground” electrodes were replaced by common mode sense (CMS) active and driven right leg (DRL) passive electrodes. The signals were pre-amplified at the electrode sites, post-amplified with DC amplifiers, and digitized at a sampling rate of 1,024 Hz (ActiView acquisition program). The signals of each electrode were referenced to the mean signal of all electrodes. Four Ag/AgCl electrodes placed near the outer canthus of each eye and under/over the left eye orbit allowed us to control blinks and horizontal and vertical eye movements. We primarily based our analyses on the P_1_N_1_ wave extracted from the SEP evoked by the tactile stimulation induced by the weight transfer (active task) or the platform translation (passive task). Consistent with studies on cortical potentials evoked by lower limb stimulation (23, 47), the SEPs were found to be maximal over the vertex (Cz electrode). The cortical SEP (P_1_N_1_) was obtained by averaging, for each participant, all synchronized epochs relative to the onset of the mechanical stimulus (i.e., shear force). For both the active and passive tasks, the stimulus onset was identified at the onset of the increase of the lateral shear forces (see Figure 1B). The average amplitude computed 50 ms prior to this onset served as the baseline. The amplitude of P_1_N_1_ was measured from peak to peak, and its latency was assessed by measuring the P_1_ latency.

The cortical sources were reconstructed using Brainstorm software [(48), freely available at http://neuroimage.usc.edu/brainstorm]. We employed the minimum-norm technique to resolve the inverse problem with unconstrained dipole orientations. The forward models were computed using a boundary element method [BEM, (49)] on the anatomical MRI Colin 27 high-resolution brain template (306,716 vertices) provided by the Montreal Neurological Institute (MNI). We opted for a model with three realistic layers (scalp, inner skull, and outer skull) which yields more accurate solutions compared to a simple three concentric sphere model (50). We used the trials of a stationary task in which the participants stood still as a baseline to compute the co-variance matrices.

Single-trial EEG data were transformed in the time–frequency domain using Morlet wavelet transforms. We used a 1 Hz central frequency [full width at half maximum (FWHM) *t*_*c*_ = 3 s], which offers a good compromise between temporal and spectral resolutions (51). The analyses of the time–frequency distribution were performed in the source space. For both types of surface, we computed the mean amplitude envelope (i.e., power) of gamma (mean 30–50 Hz, step: 1 Hz) bandwidth computed between −1 and 0 s [i.e., the planning phase of the body shift (52)]. The power was normalized with respect to the motionless baseline period (−4 to −2 s) and then averaged for each task. To control whether a change in gamma power observed between the biomimetic and the smooth surface conditions was related to the planning of the body weight transfer movement, the same analyses were performed in the passive task.

### Statistics

The behavioral and EEG data were submitted to separate analyses of variance (ANOVA). A 2 × 2 ANOVA was used for mean comparisons of shear forces and SEP with the support surface (biomimetic, smooth) and task (active or passive) as intra-participant factors. Significant effects (statistical threshold of *p* < 0.05) were further analyzed using Tukey *post-hoc* tests. A paired *t*-test was used when necessary. We assessed the effect of the type of surface on the topography of the normalized gamma-band power (30–50 Hz) computed during the preparation phase of the body weight transfer [i.e., by contrasting the source of gamma-band power estimated in the biomimetic and smooth surface conditions (significance threshold *p* < 0.05, Bonferroni correction test for multiple corrections)].

## Results

### Effect of the surface on the preparation phase of the body weight transfer

**Figure 3:**
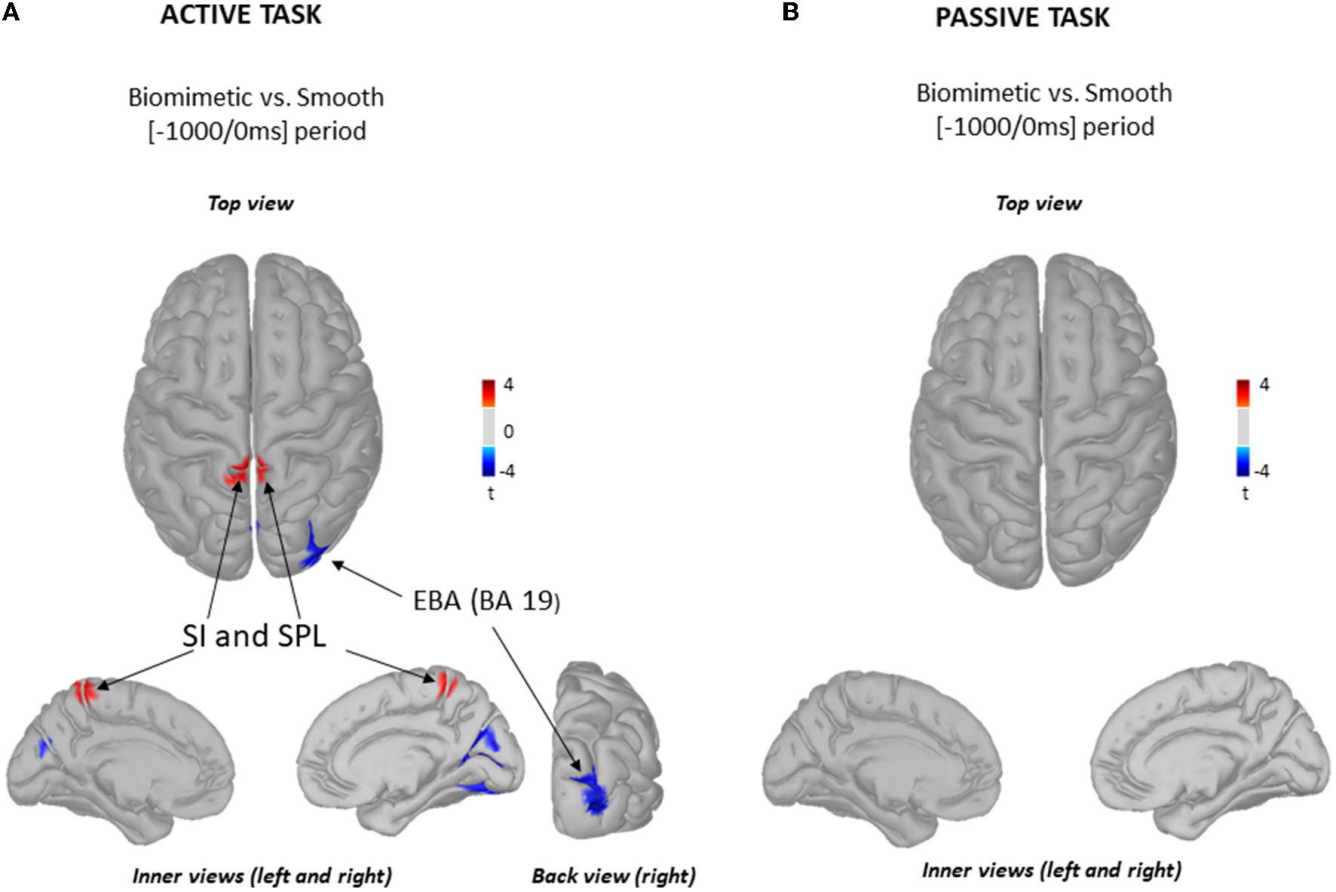
Source localization of gamma (30–50 Hz) frequency band oscillations. Statistical source localization maps for biomimetic vs. smooth contrast in the active task **(A)** and in the passive task **(B)**. Significant *t*-values (*p* < 0.05, *n* = 25) of the source localization were shown for the primary somatosensory areas (SI), superior parietal lobule (SPL), and extrastriate body area (EBA) during the time window from −1,000 ms to the onset of the mediolateral shear forces. Sources are projected on a cortical template (MNI's Colin 27). For each contrast, we display the top, left, and right inner cortical views. The back view of the right hemisphere for biomimetic vs. smooth contrast is displayed solely for the active task **(A)**.

Since the body weight transfer is prepared well in advance of its execution (52), we analyzed the cortical processing of sensory information over the 1,000-ms period preceding shear force production (Figure 3). The statistical cortical maps (Figure 3A) revealed significantly greater power (warm color) in gamma-band oscillations (30–50 Hz) in the primary somatosensory cortex (SI) and superior parietal lobule (SPL) when standing on the biomimetic than on the smooth surface. Greater activity of the extrastriate body area (EBA) in the occipital cortex (BA19, cold color) was observed when participants stood on the smooth surface. For comparison, the topography of gamma power was compared between the biomimetic and the smooth surface conditions in the passive task, which did not involve preparation for a body weight shift. For these analyses, we used the same time window as that used in the active task with respect to the onset of the shear forces, which were passively generated by the platform's translation in the passive task. The statistical source map did not reveal any significant difference between the gamma power computed in the biomimetic and smooth surfaces (Figure 3B).

### Somatosensory potentials evoked by the feet/surface interaction

**Figure 4:**
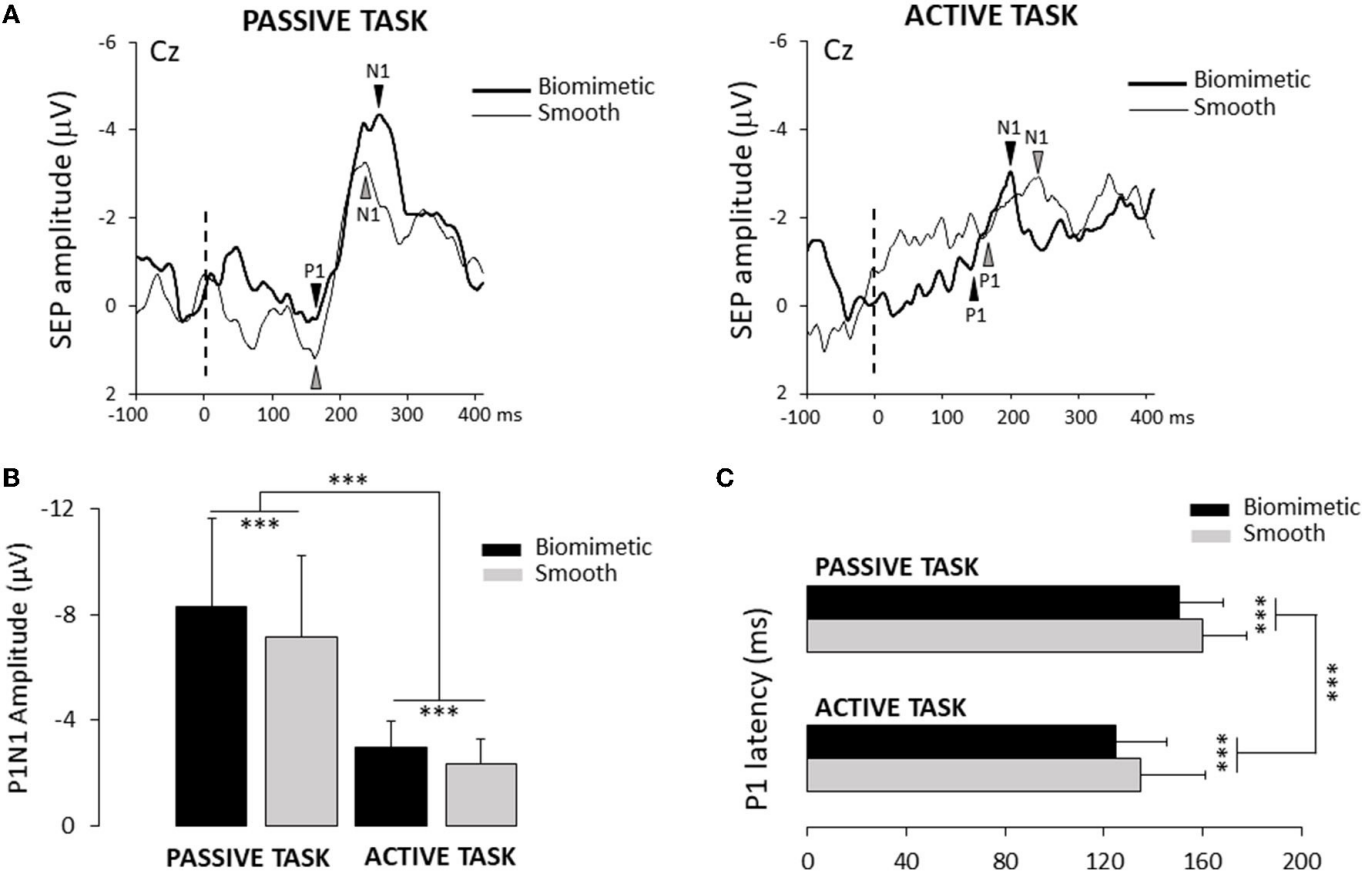
**(A)** Average of one participant of the SEP recorded over the Cz electrode for the two surfaces (biomimetic and smooth) and two tasks (passive and active). The broken line indicated the onset of the leftwards shear force. **(B)** Mean amplitude of the average P1N1 SEP on the two surfaces (biomimetic and smooth) and two tasks (passive and active). Error bars represent standard deviation across participants, ***p < 0.001. **(C)** Mean P1 latency for all participants. Error bars represent standard deviation across participants, ***p < 0.001.

To assess whether the surface and/or the task altered the response of the somatosensory areas to plantar sole cutaneous stimulation, we compared the amplitude of the P1N1 between surfaces (i.e., biomimetic and smooth) and tasks (i.e., active and passive, Figure 4A). The ANOVA showed significant surface (*F*_1, 24_ = 67.47, *p* = 1.98^*^10^−8^) and task (*F*_1, 24_ = 15.78, *p* = 5.7^*^10^−4^) effects with no significant interaction between the surface and the task (*F*_1, 24_ = 1.83, *p* = 0.19). The amplitude of the P_1_N_1_ was greater when standing on a biomimetic than on a smooth surface and greater in the passive than in the active tasks (Figure 4B). However, contrary to our predictions (i.e., less movement-related sensory gating for the biomimetic surface in the active task), the percentage of decrease of the SEP amplitude (i.e., gating) between the passive and the active tasks did not differ between the surfaces (*t*_24_ = −1.76; *p* = 0.09). On average, the overall movement-related sensory suppression was 61.4 ± 18%. The surface (*F*_1, 24_ = 19.86, *p* = 1.65^*^10^−4^) and the task (*F*_1, 24_ = 24.16, *p* = 5.2^*^10^−5^) had significant effects on P1 latency, but the ANOVA did not reveal significant task × surface interaction (*F*_1, 24_ = 0.002, *p* = 0.96). The P1 latency was shorter when participants were standing on a biomimetic (125 ± 21 ms and 150 ± 18 ms for active and passive tasks, respectively) than when they were standing on a smooth surface (respectively, 135 ± 26 ms; 160 ± 18 ms for active and passive tasks) (see Figure 4C).

We compared P1 latency and the time at which head acceleration reached the vestibular threshold during the translation to determine whether the vestibular inputs evoked by head acceleration could have been at the origin of early changes in brain activity (i.e., P1). Paired *t-*tests showed that P_1_ latencies significantly preceded vestibular stimulation onset for both surfaces in the active and passive tasks (see Table 1). This indicates that the SEP was more likely evoked by tactile inputs originating from the skin–surface interaction than by vestibular inputs. Note that the participants complied with the instruction to remain still before the task-induced changes in the shear forces as attested by head accelerometric analyses (see Figure 2C).

**Table 1:** Mean latencies of all participants (*n* = 25) and standard deviation across participants (SD) for P_1_, N_1_ and the time when the head reached the vestibular threshold as a function of the tasks and surfaces on which participants were standing.

**Biomimetic**				**Smooth**			
**Vestibular threshold**							
**Passive task**		**Active task**		**Passive task**		**Active task**	
248 ms (±34)		167 ms (±59)		233 ms (±23)		163 ms (±58)	
**P1**	**N1**	**P1**	**N1**	**P1**	**N1**	**P1**	**N1**
150 ± 18 ms	211 ± 24 ms	125 ± 21 ms	168 ± 33 ms	160 ± 18 ms	215 ± 22 ms	135 ± 26 ms	167 ± 33 ms
***T*** **-test**							
*t* = 17.75; *p* < 0.001	*t* = 5.14; *p* < 0.001	*t* = 3.37; *p* = 0.003	*t* = −0.09; *p* = 0.92	*t* = 15.45; *p* < 0.001	*t* = 4.94; *p* < 0.001	*t* = 2.07; *p* = 0.04	*t* = −0.28; *p* = 0.78

The paired *t-*test corresponds to the comparison between P_1_ or N_1_ and the vestibular threshold.

In addition to the movement-related sensory gating, the smaller SEP amplitude observed for the active task relative to the passive task could partly stem from the weaker feet-surface interaction. The impulse (i.e., the integral of the shear force over a time interval) was analyzed over a short 100-ms period (starting from the onset of the lateral shear forces change) prior to P1 SEP for all participants, surfaces, and tasks. The results showed that it was significantly more vigorous for the passive task (*F*_1, 24_ = 244; *p* = 4.43^*^10^−14^) than for the active task, but the ANOVA did not reveal a significant surface effect (*F*_1, 24_ = 1.47; *p* = 0.23). The ANOVA revealed that the interaction task x surface just reached the conventional level of significance (*F*_1, 24_ = 4.57; *p* = 0.04). However, Tukey's pairwise comparisons failed to explain this marginal global reciprocal influence of task and surface factors [see (53) for a discussion on this issue].

**Figure 5:**
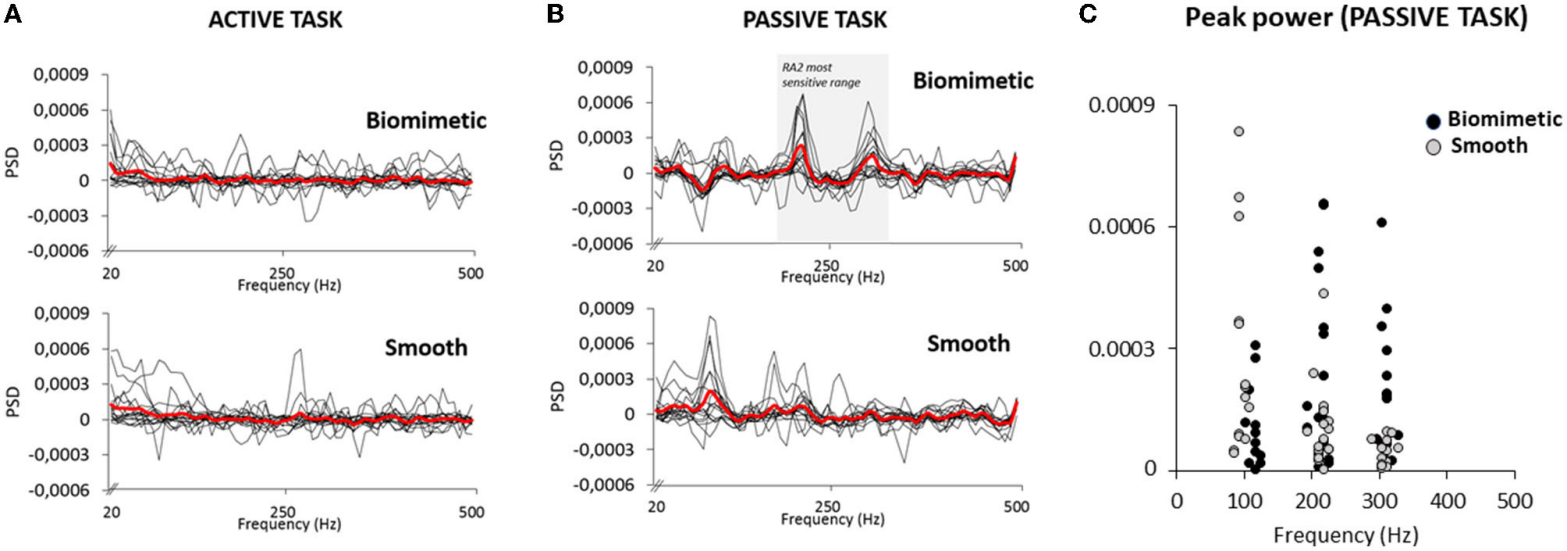
Power spectral density (PSD) of the accelerometer signal for 17 participants in active task **(A)** and the passive task **(B)**. The thick red lines represent the averaged PSD of all participants. The shaded area (**B** top panel) corresponds to the RA 2 (i.e., Pacinian corpuscles) most sensitive range. **(C)** Peak PSD for all participants exhibited at their own frequency in the passive task. Note that the peak observed around 100 Hz was likely due to the deactivation of the electromagnet to trigger the platform translation.

The vibrations arising from the feet–surface interaction were also investigated to get an estimate of the cutaneous stimulation during the active and passive tasks, in both the biomimetic and smooth surface conditions. In the active task, no characteristic vibration signature emerged, the mean PSD being close to 0 in all frequencies for both tested surfaces (Figure 5A). In the passive task, however, clear and pronounced PSD peaks were observed at ~200 and ~300 Hz for most participants with the biomimetic surface (see Figure 5B). Several participants also showed PSD peaks at these frequencies with the smooth surface, but with markedly less power (see Figure 5C). These results suggest greater stimulation of vibratory-sensitive mechanoreceptors in the passive than in the active task. To determine whether the PSD significantly differed, in the passive task, between the biomimetic and smooth surfaces, we submitted to separate *t*-tests the maximal PSD values computed for each participant and surface, between 190 and 230 Hz (referred as 200 Hz frequency) and between 290 and 330 Hz (referred as 300 Hz frequency). The results from these *t*-tests showed that the PSD was significantly greater for the biomimetic than for the smooth surface conditions at both 200 Hz (*t*_16_ = 3.06; *p* = 0.007) and 300 Hz (*t*_16_ = 3.46; *p* = 0.003).

## Discussion

The aim of this study was to determine whether the well-known movement-related sensory suppression during self-generated motor actions is partly alleviated when foot cutaneous stimulation is shaped to be relevant for balance control [e.g., FIV that enhances subcutaneous stress vibrations in a way that facilitates their processing by the Pacinian corpuscles channel (54)]. Designed with a texture inspired by the characteristics of human mechanoreceptors and dermatoglyphics (i.e., toeprints), the biomimetic surface used in the present study has been found to enhance the cortical processing of foot cutaneous information and to decrease standing task difficulty as compared to a smooth surface (20). In Sutter et al.'s study (20), foot receptors were stimulated by translating the supporting surface on which the participants were standing motionless. The same task was used here as a baseline for controlling for the movement-related sensory suppression when the cutaneous stimulation was evoked by a voluntary body weight shift (i.e., self-generated motor action) while the participants were standing on either a smooth or a biomimetic surface.

Contrary to our predictions, when executing a body weight transfer on a biomimetic surface, the well-known movement-related gating of somatosensory information was not alleviated as shown in other studies when information is relevant to the behavior (12, 55–58). For both surfaces, the sensory suppression led to a 60% reduction when compared to the SEP amplitude measured in the passive task. This sensory gating is then comparable to the sensory gating observed during electric stimulation of the tibial nerve during walking [as compared to standing, e.g., 64–69% in (3)].

Although the amount of sensory suppression was similar between the surface textures when participants performed a voluntary weight shift, the SEP amplitude remained greater when standing on the biomimetic surface in both active and passive tasks. The greater amplitude of the SEP suggests augmented stimulus intensities for activating somatosensory cortical responses. Unexpectedly, the biomimetic surface resulted in greater FIV only in the passive task wherein two pronounced PSD peaks were observed at ~200 and ~300 Hz. These frequency peaks have been observed by Scheibert et al. (39) when sliding a fake finger with ridges on a surface, but not when sliding a smooth fake finger. Based on the microneurography literature, these frequencies targeted the preferred frequencies of Pacini skin mechanoreceptors responses. Indeed, the Pacini corpuscles (RA2) respond to mechanical transitions and vibrations with the highest sensitivity around 300 Hz (59) and respond to very small skin motion (≈10 nanometers) at 200 Hz (60).

While the greater SEP observed in the active task when standing on a biomimetic surface could not be explained by an increase in skin vibrations, the augmented contribution of other peripheral stimuli with this surface cannot be dismissed [e.g., lateral skin stretch stimulating slow-adapting type II, (41)]. Indeed, the biomimetic surface with circular patterns, similar to finger- or toeprints, could enhance the encoding of variations in the skin strain distributions, thanks to the multidirectional deformation of the skin in contact with the ridges of the biomimetic surface. This has been observed by Prevost et al. (54) when the fingerprints are oriented perpendicular to the scanning direction. A spatially variable distribution of the stress–strain fields in the skin, following the surface ridges along the overall spatial directions, could also increase the detectability of its variation and provide additional information on the directionality of the causes (e.g., direction of platform/body motion). In the passive task, the shear stresses between skin and surface were directly generated by the tangential rigid motion of the platform, inducing local sliding (i.e., relative motion) between skin and surface asperities, which are at the origin of FIV. Inversely, in the active task, the load was applied by the weight redistribution operated by the participant, without a direct tangential relative displacement between the foot sole and the platform (i.e., lower impulse for the shear force), explaining the FIV's low-frequency content.

The fact that the sensory gating was not alleviated in the active task may also suggest that the biomimetic surface enhanced the sensory processing at an earlier stage, i.e., during the preparation period of the body weight shift. Indeed, previous studies have shown that somatosensory information from the lower limb is crucial for shaping, before their execution, the postural adjustments (i.e., shear forces) responsible for initiating the body weight shift (22, 24, 25). This sensory information is thought to have less importance after the postural adjustment onset (24). In line with this interpretation, we observed increased gamma rhythms in somatosensory and SPL areas during the preparation period of the body shift when the participants stood on the biomimetic surface. An increase in gamma power reflects cortical activation (61, 62). Our spectral analyses of the EEG activity, therefore, corroborate the results of previous studies supporting the idea that gamma oscillations correlate better with movement preparation than with movement execution (61, 63–65). This was also confirmed in the present study by the absence of surface-modulation of gamma activity in the passive task, wherein no movement was prepared. It should be noted that the translation of the supporting surface evoked a postural reaction (on average 126 ± 15 ms after the platform started to move) that helped the participants keeping their center of mass within their base of support (66, 67). The fact that we did not observe increased gamma power when the participants stood on the biomimetic surface in the passive task despite the presence of self-generated movements (as in the active task), albeit without a preparation period, rules out the possibility that the gamma modulation observed in the biomimetic/active condition was linked to movement execution *per se*.

A striking result of the present study is that the amplitude of the gamma activity was spatially modulated as a function of the supporting surface during the preparation period. A topographical gamma expression over centroparietal regions (SI and SPL) was observed when standing on a biomimetic surface, whereas it was observed over occipitotemporal areas (EBA) with the smooth surface. Overall, these results suggest that gamma-band oscillations play an important functional role in differentially processing tactile stimuli from the foot soles. The increased power of gamma oscillations in SI and SPL for the biomimetic surface is consistent with the hypothesis that gamma may modulate the gain of incoming tactile information in preparation for the upcoming execution of the body weight transfer. Supporting this interpretation, Palmer et al. (33) showed a high correlation between an increase gamma ERS during the preparation period and increased accuracy of upcoming tactile perception during a force-matching paradigm. Interestingly, this interpretation is also supported by studies showing that gamma-band synchronization can promote the transfer of relevant information between different regions of the cortex (68, 69). The increased gamma activity observed over S1 and SPL cortical areas accounts for this possibility as both cortical areas receive tactile afferents. Indeed, the somatosensory nature of these two areas has been suggested by neuroanatomical studies (70–72). Interestingly, the different nuclei of the “somatosensory thalamus”, conveying tactile afferents from the periphery to cortical areas, project in different combinations and with different densities directly to the postcentral somatosensory areas 3b, 1, or 5 (part of the SPL) which respond to cutaneous stimuli (72). Area 5, which is traditionally considered to be a high-order area positioned after the information process in areas 3b and 1, was identified by Impieri et al. (71) to have direct projections from nuclei composing the “sensory thalamus” and to receive strong afferences from the distal part of the limb (in particular the legs). Based on electrophysiological and neuroanatomical studies, gamma activity in both S1 and SPL could constitute the neural underpinning of the facilitation process of relevant tactile information on foot skin–surface interaction during the preparation period of motor execution. By contrast, when the relevance of tactile cues decreases as when participants stood on the smooth surface, the EBA was engaged, likely to estimate the current state of the body in space, a necessary requirement for specifying a motor plan (73). The localization of gamma activity over the EBA points to a visual representation of body and limb position in space (74, 75) probably used when there is low confidence in relying on sensory cues (here, irrelevant cutaneous inputs).

## Data availability statement

The raw data supporting the conclusions of this article will be made available by the authors, without undue reservation.

## Ethics statement

The studies involving human participants were reviewed and approved by Research Ethics Committee CERSTAPS (IRB00012476-2021-09-12-140). The patients/participants provided their written informed consent to participate in this study.

## Author contributions

CS conceived the idea, designed the method, performed experiments, analyzed the data, and wrote the manuscript. AM conceived the idea, designed the method, and contributed to data acquisition. LF, FM, and JB contributed to the analysis, interpretation of data, and the writing and critical revision of the study. LM conceived the idea and contributed to data acquisition, the analysis, interpretation of data, and the writing and critical revision of the study. All authors contributed to the article and approved the submitted version.
